# Recurrent Cranial Chondrosarcoma: A Case Report

**DOI:** 10.30699/IJP.2021.532878.2663

**Published:** 2021-12-15

**Authors:** Elham Nazar, Shabnam Mashhadi, Golnaz Moradi

**Affiliations:** 1 *Department of Pathology, Sina Hospital, Tehran University of Medical Sciences, Tehran, Iran*; 2 *Department of Radiology, Sina hospital, Tehran University of Medical Sciences, Tehran, Iran*

**Keywords:** Cranial, Chondrosarcoma, Recurrence

## Abstract

Chondrosarcoma of the cranium is a rare malignancy. The result of treatment is challenging to assess because the slow-growing rate means that there is a long interval previously discovering the recurrence and last long time to diagnosis of recurrence. This report describes a 38-year-old man who presented with a generalized seizure 2 months before his referral. The patient underwent excisional surgery. The histological examinations revealed a cartilage developing tumor compatible with chondrosarcoma. The radiologic and histologic correlation established the diagnosis. But, the patient had two episodes of recurrence after surgery. We determined that intracranial chondrosarcoma must be comprised in the differential diagnosis of a mass with calcification on cranial imaging. Accurate diagnosis is obligatory for supplementary patient managing, and a recurrence is more common in patients only treated by surgery.

## Introduction

Chondrosarcoma is the second common primary malignancy of bone, ascending from chondrocytes origin through the axial and appendicular skeletal system (1). The pathogenesis of this malignancy is vague; it has been suggested that intracranial chondro-sarcoma progresses from the chondrocytes in endoch-ondral cartilage remnant existent in the skull base (2). Chondrosarcoma is a rare malignancy that occurs about 0.15% of totally primary intracranial masses and 6% of total skull base masses. It can be comparable to chondroma, and it is repeatedly misdiagnosed as such (3). Intracranial chondrosarcoma is an extraordinary malignancy, estimating almost one in 1000 intracranial neoplasms. These tumors are usual in the axial and appendicular skeletal system and occurrence in intracranial is a problematic and oncological issue (4). It is necessary to precisely discriminate chondrosarcoma from other intracranial neoplasms such as enchondroma and myxoid tumors (chordoma and chondromyxoid fibroma) in order to define their curative and predictive consequences (5). Herein, we report chondrosarcoma initiating from the dura mater in the frontoparietal area to increase the alertness about the intra-cranial chondro-sarcomas. Furthermore, this case presents several local recurrences; this experience has rarely been described in intracranial cases.

## Case report

**Fig. 1:**
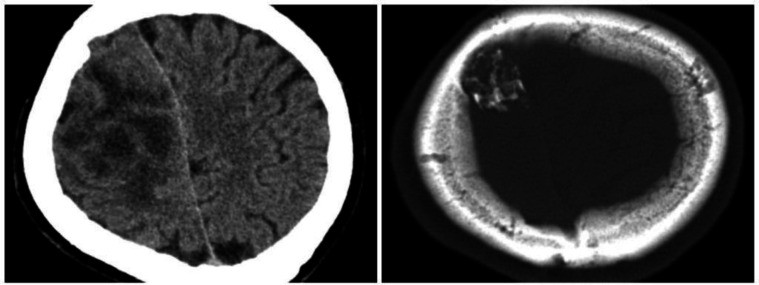
Preoperative non-contrast computed tomography revealed a heterodense mass with a calcified component in the right frontoparietal region

**Fig. 2:**
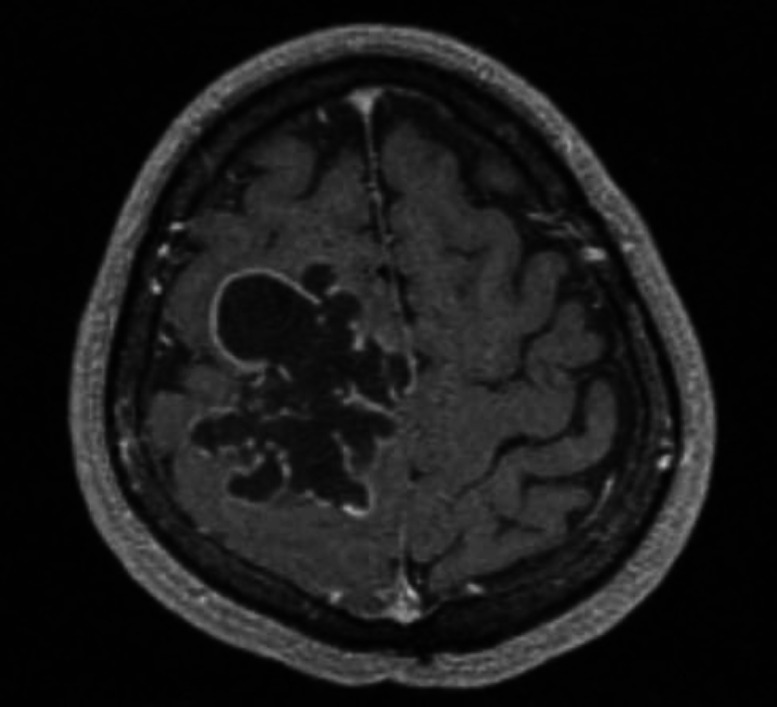
Preoperative non-contrast T1-weighted images revealed a lobulated hypointense mass with peripheral rim-like enhancement

A 38-year old male was referred to the department of neurosurgery in Sina hospital affiliated with Tehran University of Medical Sciences, Tehran, Iran, with a history of generalized seizure from 2 months before his referral. At first, outpatient examination findings were unremarkable. The patient’s family history and past medical history were none. General examination was negative. Routine laboratory investigations were the normal limit. The patient underwent a brain computed tomography (CT) scan, which showed a heterodense mass with a calcified component in the right frontoparietal region, causing erosion of frontal bone on non-contrast imaging (Figure 1). The post-contrast T1-weighted images showed a lobulated, hypointense mass with peripheral rim-like enhancement (Figure 2).

**Fig. 3:**
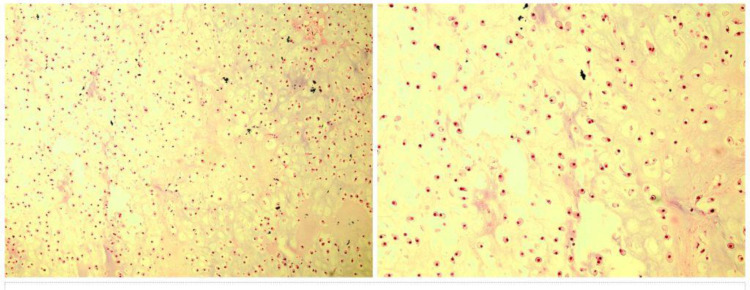
Histopathologic examination shows neoplastic tissue consisting of a well-differentiated chondroid component with lobular architecture, compatible with well-differentiated classic chondrosarcoma (Grade 1) (H&E, x100 and x200, respectively)

Diagnosis needed histopathologic investigation. The patient underwent excisional surgery. Through the pathology description, it was discovered that neoplastic tissue consisted of a well differentiated chondroid component with lobular architecture. Also, there were no mitotic figures and necrosis (Figure 3). The final pathology findings established that the brain lesion was well-differentiated classic chondrosarcoma (Grade 1). No evidence of tumor spread was found in other sites. The patient received no adjuvant therapy. Follow-up examination showed recurrence on imaging after 6 months, and the patient underwent further surgery. Again, 2 years later, the patient experienced a recurrence. After the latest surgery, the patient was a candidate for adjuvant radiation therapy. Surprisingly, all resected lesions in any recurrence were well-differentiated classic chondrosarcoma (Grade 1). After 6 months of follow-up examinations from the recent surgery, no recurrence was observed, and the patient is still asymptomatic.

## Discussion

The occurrence of benign bone and cartilage neoplasms is mainly unclear due to the lack of reporting as well as failure to present clinically (6). Three subtypes of chondrosarcomas have been categorized according to histology appearance; classic chondrosarcoma, mesenchymal chondrosarcoma, and myxoid chondrosarcoma (7). The mesenchymal subtype has a more malignant potential with an increased tendency for recurrence, metastasis, and high vascularity (2). But, our case with the classic type of chondrosarcoma had several recurrence episodes. The metastasis from chondrosarcoma to respiratory organs is a common happen (8). Also, their close correlation with cranial nerves and main vessels of the head and neck, complete surgical resection frequently damaged them (9). Cranial chondrosarcoma is challenging to manage due to its unreachable location.

The current basis of handling is surgical resection which is well surveyed by adjuvant radiation therapy. Chemotherapy has been predominantly ineffective for chondrosarcoma because there is a deficiency of targeted therapies (10). Re-growth of tumor after surgery occurred in 53% of the patients (about after 32 months). In our case, the first recurrence was observed after 6 months which cannot rule out the probability of incomplete resection. There is treatment-associated morbidity and mortality due to surgery and radiotherapy, about which the patients must be alerted when proposing these therapies (11). Histology subtype, prior treatment (surgery or radiation therapy), and amount of tumor resection and adjuvant radiation therapy after surgery have all been shown to affect patient results. However, local recurrence is considered the most significant indicator of mortality in these patients (12). Mortality at 5 years is meaningfully higher in tumors of higher grade, or of the mesenchymal subtype, or who had been treated by surgical resection only (13). The five-year recurrence frequency among all patients is 22%, with an average follow-up of 60 months and an average disease-free interval of 16 months. Tumor recurrence is more common in patients who are only treated by surgery or have a mesenchymal subtype of chondrosarcoma (2), which in our case, despite the classic type of chondrosarcoma, the patient received no adjuvant therapy and had several recurrences. Also, chondrosarcoma of the cranial is an infrequent malignancy that can be similar to chordoma, and in detail, it is misdiagnosed repeatedly as such. This has significant clinical consequences, as while treated with aggressive curative plans, chondrosarcoma has a considerably better prognosis than chordoma (12). Although definite preoperative diagnosis for the skull base chondrosarcoma is difficult, approaches for diagnosis and treatment devoid of any problem are serious. 

## Conclusion

Chondrosarcoma in the cranial area is rare, but they must be careful in the differential diagnosis of various cranial masses. Difficulties in the differential diagnosis might occur in connection with an extraordinary setting. They reveal common unexpected clinical signs that, to conclude, lead to inadequate treatment. It is our hope that with such reports, we are able to reduce the effects of individual surgeons and individual foundation bias on the outcome of these patients, in that way making a more independent protocol for physicians and patients in the future management of these tumors.

## Conflict of Interest

None.

## Funding

None.
